# Imaging features of sentinel lymph node mapped by multidetector-row computed tomography lymphography in predicting axillary lymph node metastasis

**DOI:** 10.1186/s12880-021-00722-0

**Published:** 2021-12-15

**Authors:** Xiaochan Ou, Jianbin Zhu, Yaoming Qu, Chengmei Wang, Baiye Wang, Xirui Xu, Yanyu Wang, Haitao Wen, Andong Ma, Xinzi Liu, Xia Zou, Zhibo Wen

**Affiliations:** 1 Department of Radiology, Zhujiang Hospital, Southern Medical University, 253 Gongye Middle Avenue, Haizhu District, Guangzhou, 510282 Guangdong China; 2 Department of Breast Surgery, Zhujiang Hospital, Southern Medical University, 253 Gongye Middle Avenue, Haizhu District, Guangzhou, 510828 Guangdong China

**Keywords:** Breast cancer, Multidetector-row computed tomography lymphography, Sentinel lymph node, Axillary lymph node metastasis, Cortical thickness

## Abstract

**Introduction:**

Accurately assessing axillary lymph node (ALN) status in breast cancer is vital for clinical decision making and prognosis. The purpose of this study was to evaluate the predictive value of sentinel lymph node (SLN) mapped by multidetector-row computed tomography lymphography (MDCT-LG) for ALN metastasis in breast cancer patients.

**Methods:**

112 patients with breast cancer who underwent preoperative MDCT-LG examination were included in the study. Long-axis diameter, short-axis diameter, ratio of long-/short-axis and cortical thickness were measured. Logistic regression analysis was performed to evaluate independent predictors associated with ALN metastasis. The prediction of ALN metastasis was determined with related variables of SLN using receiver operating characteristic (ROC) curve analysis.

**Results:**

Among the 112 cases, 35 (30.8%) cases had ALN metastasis. The cortical thickness in metastatic ALN group was significantly thicker than that in non-metastatic ALN group (4.0 ± 1.2 mm vs. 2.4 ± 0.7 mm, *P* < 0.001). Multi-logistic regression analysis indicated that cortical thickness of > 3.3 mm (OR 24.53, 95% CI 6.58–91.48, *P* < 0.001) had higher risk for ALN metastasis. The best sensitivity, specificity, negative predictive value(NPV) and AUC of MDCT-LG for ALN metastasis prediction based on the single variable of cortical thickness were 76.2%, 88.5%, 90.2% and 0.872 (95% CI 0.773–0.939, *P* < 0.001), respectively.

**Conclusion:**

ALN status can be predicted using the imaging features of SLN which was mapped on MDCT-LG in breast cancer patients. Besides, it may be helpful to select true negative lymph nodes in patients with early breast cancer, and SLN biopsy can be avoided in clinically and radiographically negative axilla.

## Background

Recently, a large population-based study reported that an increasing global incidence of breast cancer and there are about 645,000 premenopausal and 1.4 million postmenopausal breast cancer cases were diagnosed worldwide [1]. Breast cancer may often be associated with axillary lymph node (ALN) metastases, and ALN status is one of the most important predictor of overall recurrence and survival in patients with breast cancer. Accurate assessment of ALN disease burden is vital in staging breast cancer, which guides multidisciplinary treatment decision making nowadays [2, 3]. Sentinel lymph node (SLN) biopsy (SLNB) is now widely used as the gold standard for axillary staging in clinically node-negative breast cancer in National Comprehensive Cancer Network (NCCN) guidelines [4], which reduces the complications of lymphedema and other arm morbidity to a certain extent compared with axillary lymph node dissection (ALND) [5].

Assessment of ALN disease involvement is currently considered to be the most important role of axillary imaging [2]. In clinical practice, conventional imaging techniques for ALN status assessment include mammography (MMG), ultrasonography (US) and magnetic resonance imaging (MRI) [6]. Axillary level I lymph nodes (LNs) are visible at routine MMG in 50% of patients [7], but limited visualization makes it unreliable to assess ALN [8]. US is the primary modalities to evaluate ALN, nonetheless, results may vary according to operator mainly [9]. A retrospective study has shown that US cannot distinguish between localized and advanced ALN disease when the result is positive [10], which may lead to over-treatment under the Z0011 standard since 40–70% patients had the SLN as the only site of nodal metastasis [11]. Indeed, some medical institution have abandoned preoperative axiilary US in patient with negative finding on physical examination to avoid triaging those women with positive ALN directly to ALND [12, 13]. ALNs with standard breast MRI revealed comparable performance to that of US [14]. Although MRI has dedicated breast coil, its ability to show the complete axilla is limited, and cardiac motion artifacts can occasionally block ALN, especially in leve II and III LNs [15]. The indications of MRI mainly include clinically staging of breast cancer, screening of high-risk populations and efficacy evaluation of neoadjuvant chemotherapy [16], However, it may be more commonly used for imaging and detection of primary cancers. In addition, MRI is time-consuming and expensive, making it not widely used in clinical practice [15].

Many previous studies have shown that multidetector-row computed tomography (MDCT) can be used to evaluate regional LNs [17–19]. High-resolution helical CT could show not only eccentric or irregular cortical thickening in metastasis LN, but detect extracapsular LN extension [20], which is a potent imaging tool for predicting ALN metastasis. Besides, Chest CT can be used to evaluate axillary nodes in patients with advanced breast disease and showed better diagnostic value for visualizing level III LNs, interpectoral nodes, and extensive nodal involvement [21]. Chen et al. had shown that MDCT is an effective imaging tool for predicting ALN metastasis [22] Preoperative multidetector-Row computed tomographic lymphography (MDCT-LG) can be used to locate SLN in patient with early breast cancer [23], because the tumor cells almost always invade ALN sequentially and usually starting with SLN [24, 25]. Moreover, studies have shown that MDCT-LG in addition to being reliable navigation instrument for SLN biopsy (SLNB) that helps surgeons quickly find the SLN, reduce surgical time and improve the accuracy of the SLNB, it may also be helpful in determining the scope of LNs cleaning [26], The anatomic morphology of lymphatic vessels and SLNs can also be understood preoperatively, which is beneficial to improve the success rate of SLNB surgery [27]. Nakagawa and Ashiba et al. used MDCT-LG to diagnosed SLN metastasis before surgery as well, But neither took into account the thickness of the LN cortex [28, 29].

The purpose of our study was to assess the value of MDCT-LG determined SLN imaging features in predicting ALN status.

## Methods and materials

### Patients enrollment

The study was approved by the local Institutional Review Board, and because its nature of retrospective study, the requirement of obtaining informed consent was waived. Breast cancer patients who underwent MDCT-LG and had a clear display of SLNs and subsequent surgery between January 2019 and March 2021 were included in our study. The inclusion criteria were: (1) Women aged 18 years or older; (2) Images can clearly locate sentinel lymph nodes; (3) First diagnosis of breast cancer; (4) Breast cancer was pathologically confirmed by surgery or biopsy; The exclusion criteria included: (1) patients with incomplete pathological results; (2) patients were diagnosed with distant metastasis; (3) patients who had undergone tumor resection or neoadjuvant chemotherapy prior to MDCT-LG; (4) Lactation patient; All the patients received surgery for axillary staging, included SLNB, ALND or both. The number of lymph nodes resected were recorded, with subsequent pathological examination and confirmation by two pathologists. Clinicopathological characteristic (including age, sex, tumor size, histology, and ALN metastasis status) were collected.

### MDCT-LG

All images were obtained with a 64-detector row CT scanner (Brilliance 64, Philips, Netherlands). Patients were placed in a supine position with their arms positioned in a cranial direction. Local anesthesia was performed by subcutaneous injection of 4 ml lidocaine (0.02 g/ml) with a 5-ml disposable sterile syringe (Zhejiang Jinghuan Medical Supplies Co., LTD), followed by, intradermal injection of 1-ml iopamidol (370 mg/ml, Obilol, Shanghai, Bolaik Xini Pharmaceutical Co.,LTD) of intradermal injection at 3, 6, 9 and 12 o’clock of areola, respectively. Finally contiguous 1-mm-thick images that included the breast and axilla were obtained after gently massaging the injection site for about 30 s. The CT scanning was performed with the following parameters: 120 kV and 250 mA, field view of 32 cm × 32 cm, matrix of field of view a 512 × 512, a section spacing of 1 mm. The number of sections were adjusted for each individual to ensure coverage of breast and axillary areas. After the CT scanning, a 3D reconstruction was performed on the Philips IntelliSpace Portal to determine the SLN and its location. The identification of the SLN was completed by a breast surgeon and a radiologist with 3 years of experience.

### MDCT-LG evaluation of the nodal status

**Fig. 1:**
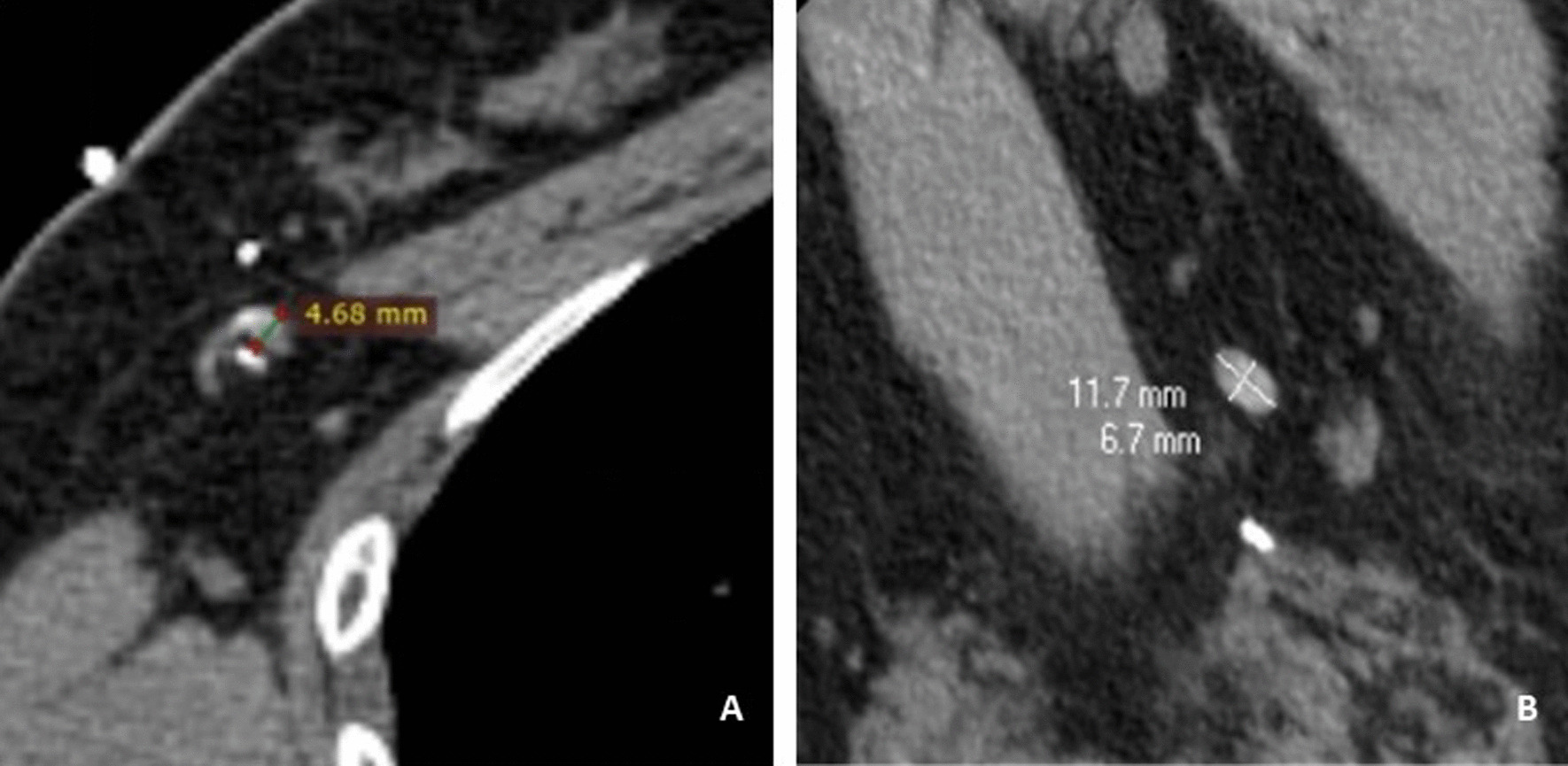
Methods of measuring long and short diameter of sentinel lymph nodes and cortical thickness. **A** The maximum layer of cortical thickness of selected sentinel lymph nodes was measured in RadiAnt DICOM Viewer (64-bit). **B** The maximum long diameter and maximum short diameter of the maximum section of the selected sentinel lymph node were measured in Philips IntelliSpace portal

MDCT-LG Evaluation was performed on the Philips IntelliSpace Portal platform with 3D resconstruction. Two radiologists (with 3 years of experience of breast-radiology) reviewed the images and measured the relevant parameters. For those with more than one SLNs, the largest one would have been selected and measured. The recorded parameters include: (1) the shape of SLN; (2) long-axis diameter; (3) short-axis diameter; (4) Ratio of long-/short-axis; (5) cortical thickness. The data measurement method is shown in Fig. 1. According to the pathological results of ALN, they were divided into metastatic group and non-metastatic group. The relationship of lymph nodes to pectoralis minor divides the axillary lymph nodes into levels I, II and III. The lymph nodes located lateral to the lateral border of the pectoralis minor are level I nodes. The axillary located between the medial and lateral boundaries of the pectoralis minor or interpectoral (Rotter’s) lymph nodes are level II nodes. The lymph nodes located medial to the medial margin of the pectoralis minor muscle and inferior to the clavicle are level III nodes [30].

### Pathology

Primary breast cancer is divided into invasive breast cancer and ductal carcinoma in situ according to pathology. T1 disease is defined as a primary tumor size of less than or equel to 20 mm; T2 disease is defined as a primary tumor size of more than 20 mm but less than or equal to 50 mm; T3 disease is defined as a primary tumor size of more than 50 mm [30]. The pathologist diagnosed each lymph node as benign or malignant.

### Statistical analysis

Continuous and categorical variables were analyzed with Mann Whitney U-test and χ2-test respectively. Logistic regression analysis (stepwise) was performed to evaluate the variates associated with ALN metastasis so that significant or marginal significant factors (*P* < 0.05) could be identified. Receiver operating characteristic (ROC) curve analysis was performed to evaluate the MDCT variates for predicting ALN metastasis. The area under the ROC curve (AUC) was evaluated for diagnostic ability. The optimal cutoff value was based on the ROC curve with Youden’s J statistic (J), J = sensitivity + specificity − 1. *P* < 0.05 was considered statistically significant. Statistical analysis was performed using SPSS statistical software (version 18.0, SPSS Inc., Chicago, IL, USA) and MedCalc Statistical Software (version 19.5.6, MedCalc Software bvba, Ostend, Belgium).

## Result

### Patients characteristic

A total of 149 cases underwent MDCT-LG and had a clear display of SLNs. Thirty-seven patients were excluded because of: (1) incomplete pathological results (n = 14); (2) Distant metastasis (n = 3); (3) tumor resection or neoadjuvant chemotherapy prior to MDCT-LG (n = 19); (4) during breast feeding (n = 1). Finally, 112 cases were enrolled in the study. A total of 108 cases (96.4%) of SLNs identified by preoperative MDCT-LG were at the level I, and 4 cases (3.6%) were at the level II of the axillary clinical group. Totally have 87 cases (78.3%) performed SLNB, 24 (20.0%) cases performed both SLNB and ALND, and 1case (1.7%) performed ALND directly.

All patients were female, with a mean age of 49.7 years (range 23–76 years). Tumor size was measured based on gross specimen after surgery, with a median tumor size of 14.5 mm (range 7–80 mm). In line with the American Joint Committee on Cancer (AJCC) 7-stage system, 53 (47.3%) were stage T1, 53 (47.3%) were stage T2, and 6 (5.4%) were stage T3. Pathological examination revealed that 108 cases (96.4%) were invasive breast cancer and 4 cases were ductal carcinoma in situ (DCIS). All patients underwent axillary surgery, 35 (30.8%) cases with ALN metastasis, including 22 cases (19.6%) with SLN metastasis alone, 12 cases (10.7%) with both SLN and non-SLN metastasis, and 1 case (0.9%) with non-SLN metastasis alone (Table 1).

**Table 1:** Patient characteristics

Characteristics	Median	No. of patients/SLN (%)
Female		112 (100.0)
Age(years)	49.7 (23–76)	
Tumor size(mm)	24.5 (7–80)	
*T* ≤ 20 mm		53 (47.3)
20 mm < *T* ≤ 50 mm		53 (47.3)
*T* > 50 mm		6 (5.4)
Histology		
Invasive breast cancer		108 (96.4)
DCIS		4 (3.6)
ALN metastasis		35 (100.0)
Only SLN		20 (17.9)
Only non-SLN		3 (2.8)
Both SLN and non-SLN		12 (10.7)

### MDCT-LG variables of sentinel lymph nodes

As shown in Table 2, the long-axis diameter and short-axis diameter of metastatic SLN were significantly longer than that of non-metastatic SLN (14.6 ± 6.2 mm vs.11.1 ± 3.3.mm, *P* < 0.001; 10.7 ± 4.2 mm vs. 6.2 ± 2.0 mm, *P* < 0.001). The average ratio of long-/short-axis of metastatic SLN was significantly shorter than that of non-metastatic SLN (1.4 ± 0.3 vs. 1.7 ± 0.3, *P* = 0.002). The thickness of cortex in metastatic SLN was significantly thicker than that in non-metastatic SLN (4.0 ± 1.2 mm vs. 2.4 ± 0.7 mm, *P* < 0.001). No significant difference was found in the shape of SLN (*P* = 0.352) (Table 2).

**Table 2:** Comparison of SLN metastasis with MDCT-LG variables

Characteristic	ALN metastasis			*P* value
		Positive (n = 35)	Negative (n = 77)	
Long-axis diameter(mm)	11.6 ± 4.8	14.6 ± 6.2	11.1 ± 3.3	< 0.001
Short-axis diameter(mm)	7.0 ± 3.4	10.7 ± 4.2	6.2 ± 2.0	< 0.001
Ratio of long-/short-axis	1.6 ± 0.3	1.4 ± 0.3	1.7 ± 0.3	0.002
Cortical thickness(mm)	2.6 ± 1.1	4.0 ± 1.2	2.4 ± 0.7	< 0.001
Shape				0.352
Oval	92	27	65	
Round	20	8	12	

### Univariate and multivariate analysis

Univariate analysis showed that long-axis diameter, short-axis diameter, ratio of long-/short-axis, cortical thickness were significantly associated with ALN metastasis. Multivariate analysis demonstrated that cortical thickness were independent predictors for ALN metastasis (OR 24.53, 95% CI 6.58–91.48, *P* < 0.001) (Table 3).

**Table 3:** Univariate and multivariate logistic regression analysis for SLN metastasis

Variable	Cut-off value	Univariate analysis		Univariate analysis	
		OR (95%CI)	*P* value	OR (95%CI)	*P* value
Long-axis diameter(mm)	> 13.9	7.09 (2.78–18.13)	< 0.001	–	0.249
	≤ 13.9	–	–	–	–
Short-axis diameter(mm)	> 9.1	20.03 (6.81–58.92)	< 0.001	–	0.630
	≤ 9.1	–	–	–	–
Ratio of long-/short-axis	≤ 1.7	4.24 (1.58–11.38)	0.004	–	0.310
	> 1.7	–	–	–	–
Cortical thickness(mm)	> 3.3	24.53 (6.58–91.48)	< 0.001	23.50 (6.27–88.10)	< 0.001
	≤ 3.3	–	–	–	–

### Comparison of axillary lymph nodes metastasis prediction performance

**Fig. 2:**
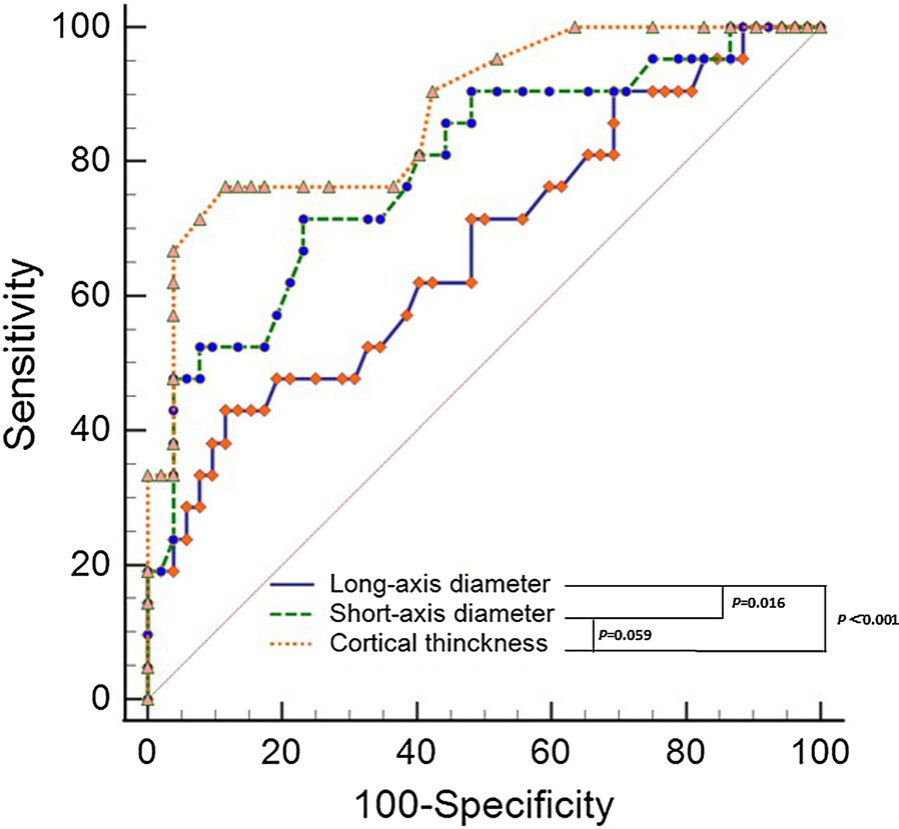
Comparison of areas under the curve of long-axis diameter, short-axis diameter, and cortical thickness

The ROC based on univariate analysis showed that 13.9 mm was the optimal cut-off value of long-axis diameter, with an AUC, sensitivity and specificity were of 0.725 (95% CI 0.633–0.805, *P* < 0.001), 54.3% and 87.0%, respectively. The optimal cut-off value of short-axis diameter was 9.1 mm, with an AUC, sensitivity and specificity were 0.801 (95% CI 0.715–0.870, *P* < 0.001), 62.8%, and 92.2%, respectively. The optimal cut-off value of ratio of long-/short-axis was 1.7, with an AUC, sensitivity and specificity were of 0.679 (95% CI 0.584–0.764, *P* = 0.004), 82.7.0% and 46.8%, respectively. The optimal cut-off value of cortical thickness was 3.3 mm, with an AUC, sensitivity and specificity were of 0.872 (95% CI 0.773–0.939, *P* < 0.001), 76.2% and 88.5%, respectively. The short-axis diameter had the highest specificity, 92.2%. The ratio of long-/short-axis had the highest sensitivity, 82.9%. (Tables 3, 4 and Fig. 2). The AUC of cortical thickness and short-axis diameter were larger than that of long-axis diameter, and it was statistically significant. The comparison of AUC between cortical thickness and short-axis diameter was not statistically significant, *P* = 0.059*.* The ratio of long-/short-axis and cortical thickness had the highest positive predictive value (82.7%) and negative predictive value (90.2%), respectively.

**Table 4:** Optimal cut-off values for diagnosis of SLN metastasis with MDCT-LG

MDCT-LG variates	Optimal cut-off value	Sensitivity (%)	Specificity (%)	AUC	PPV (%)	NPV (%)
Long-axis diameter (mm)	> 13.9	54.3	87.0	0.725	78.6	84.5
Short-axis diameter (mm)	> 9.1	62.9	92.2	0.801	64.3	79.8
Ratio of long-/short-axis (mm)	≤ 1.7	82.9	46.8	0.679	82.9	46.8
Cortical thickness (mm)	> 3.3	76.2	88.5	0.872	72.7	90.2

According to the above results of univariate analysis, three variables with *P* < 0.001 were selected for combination and their respective AUC were compared. The three variables were long-axis diameter, short-axis diameter and cortical thickness. Combined-analysis indicated that combination of both or three of the variables did not improve diagnostic specificity or sensitivity. As shown in Tables 4 and 5, the results of combination of long-axis diameter and short-axis diameter were same as those of single variable of short-axis diameter, they had the same AUC, sensitivity, and specificity. The AUC, sensitivity, and specificity of the three combined variables were the same as those of the single variable of cortical thickness.

**Table 5:** AUC comparison of combinatiorial factors

MDCT-LG variates	Sensitivity (%)	Specificity (%)	AUC
Short-axis diameter + Long-axis diameter	62.3	92.2	0.801
Long-axis diameter + Cortical thickness	76.2	88.5	0.872
Short-axis diameter + Cortical thickness	76.2	88.5	0.872
Combination of three variables	76.2	88.5	0.872

## Discussion

Our study is that we selected the MDCT-LG mapped SLN as the study subject, and using it, we can not only predict ALN metastasis, but may help select a portion of patients who can be exempted from SLNB and make treatment more personalized.

Chen et al. showed MDCT could predict ALN metastasis by observing and measuring the biggest LN in the axilla [22], but it is not clear whether the largest lymph node is the SLN. Compared with their study, we mapped SLN prior to operation, which helps clinicians determine the surgical approach of SLNB, thus shortening the surgical time and improving the accuracy of SLNB. Besides, knowing the location of SLN in advance could help surgeons to determine the approximate range of LNs resection, which is especially significant for patients with SLN at level II or III, and would not lead to SLN miss.

In terms of diagnosing lymph node status, this study demonstrated that LN metastasis of breast cancer is usually orderly, and SLN usually located at level I, which support the results of Kalli et al., namely, breast cancer cells spread progressively and orderly along the lymphatic drainage system, with level I first metastasis in most cases [30]. Both long-axis diameter and short-axis diameter of the metastasis SLN are significantly longer than that of the non-metastasis SLN, which is consistent with previous research results [31, 32]. As for the short-axis diameter, it is recommended to distinguish between benign and malignant LNs in the RECIST 1.1 [33]. In line with previous CT and MRI studies, the mean ratio of long-/short-axis of metastasis lymph node was lower than that of non-metastatic one [17, 34].

For the long-axis diameter, the ROC showed that 13.9 mm was the optimal cut-off value, and our result was close to that of Chen et al. [22], whose was 14.5 mm, but their statistical analysis indicated that long-axis diameter was not a statically significant factor, whereas, our study suggested that long-axis diameter is one of the factor to identify metastatic from non-metastatic LNs. Whether evaluating axillary or cervical nodes, short-axis diameter is commonly used by radiologist because it is a repeatable measure to predict metastasis [34]. Different sites have corresponding drainage LNs, but the recommended diagnostic criteria for short-axis diameter vary from site to site [17, 19, 35–37]. At present, there is no standard value about the optimal short-axis diameter of ALN. Our study indicated that 9.1 mm was the best short-axis diameter cut-off value. Previous researchers have used short-axis diameter ≥ 10 mm as the cut-off value for diagnosing metastatic ALN [31, 37]. In addition, the ratio of long-/short-axis on the image could also indicate ALN involvement. Liu et al.demonstrated that combination of smaller size and lymph node axial ratio can Improve CT detection sensitivity for nodal metastases in oesophageal cancer [38]. Both the two indexes of our study above are similar to those of Chen et al. [22], whose optimal cut-off values of the short-axis diameter and ratio of long-/short-axis are 9.5 mm and 1.7 respectively, and the statistical analysis results showed that there are significant differences. Although the long-axis diameter, short-axis diameter and ratio of long-/short-axis were not independent predictors, they were statistically significant in Univariate analysis, which may be caused by the insufficient number of cases.

We measured the cortical thickness of the SLN as well. Although cortical thickening is often associated with reactive lymph node hyperplasia, prospective studies of cortical thickness of ALNs in preoperative breast cancer patients have shown that the incidence of malignancy increases in proportion to cortical thickness [39]. Because malignant cells enter the LN through the subcapsular sinus in the form of afferent lymphoid deposits (local diffusion), where they grow and eventually replace locally normal lymph node structures [40]. In fact, cortical thickening of metastatic lymph nodes is often found in axillary images [39, 41]. Our results showed a significant difference in cortical thickness between the metastatic and non-metastatic groups. Moreover, cortical thickness was an independent predictor of ALN metastasis. In our study, the optimal cut-off value of cortical thickness was 3.3 mm. This is different from the results of Chen and Imai et al. whose results are 3 mm [22, 31].

**Fig. 3:**
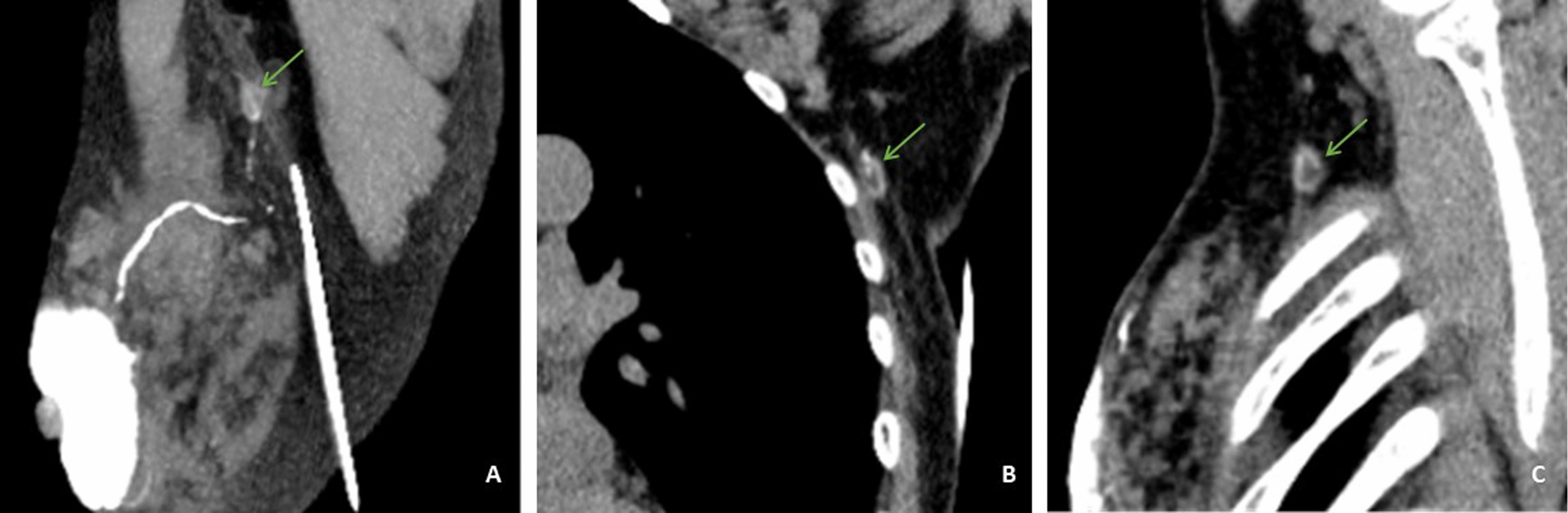
Representative multidetector-row computed tomography Lymphography (MDCT-LG) images of a 58-year-old woman with left primary breast cancer (stage pT2N0, Non-specific invasive breast cancer). **A** The MDCT-CT 3D reconstruction image showed an sentinel lymph node was in level I. **B**, **C** Sagittal and coronal MDCT-LG image showed this lymph node with oval shape, a long-axis diameter of about 12.2 mm, and a short-axisdiameter of about 5.4 mm (green arrows) and a cortical thickness about 2.2 mm (green arrows). The patient underwent sentinel lymph node biopsy, five lymph nodes were removed and proved to be pathologic negative

**Fig. 4:**
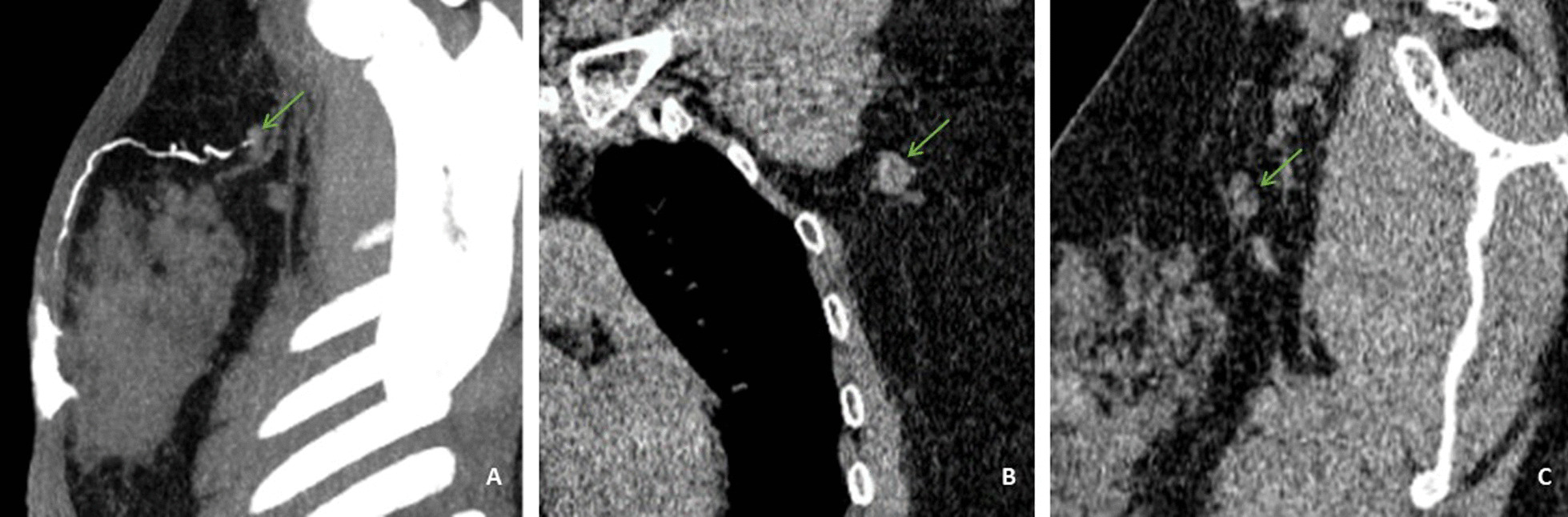
Representative multidetector-row computed tomography lymphography (MDCT-LG) images of a 59-year-old woman with left primary breast cancer (stage pT3N1, Non-specific invasive breast cancer). **A** The MDCT-CT 3D reconstruction image showed an sentinel lymph node was in level I. **B**, **C** Sagittal and coronal MDCT-LG image showed this lymph node with oval shape, a long-axis diameter of about 13.3 mm, and a short-axisdiameter of about 11.1 mm (green arrows) and a cortical thickness about 4.3 mm (green arrows). The patient underwent both sentinel lymph node biopsy and axillary lymph node dissection, a total of eighteen lymph nodes were removed, and sentinel lymph node biopsy removed seven lymph nodes, four of which proved to be pathologic positive

Ultrasound is the first choice for the assessment of ALNs and imaging guided lymph node interventional therapy [42]. Marino et al. reported sensitivity and specificity of ultrasound, standard MRI, and PET/CT in assessing ALN: 87% and 53% to 97.3%, 70% and 90%, 64% and 93%, respectively [42]. In this study, the sensitivity, specificity and AUC of the single variable of cortical thickness were 76.2%, 88.5% and 0.872, respectively. In terms of sensitivity, our results indicate that the sensitivity is not as good as that of ultrasound, but slightly better than that of PET/CT and standard MRI. In terms of specificity, our results are very close to PET/CT, but slightly inferior to MRI. Compared with standard MRI, using dedicated axillary protocols can increase the sensitivity and specificity to 84% and 95%, respectively, but they require additional scanning time and are not feasible in clinical practice [43]. Overall, SLN measured with more parameters, including cortical thickness < 3.3 mm, Short-axis diameter < 9.1 mm, and Ratio of long-/short-axis ≥ 1.7, were more likely to be negative lymph nodes and vice versa (Figs. 3, 4). In our view, when the results tend to be negative and the volume of non-SLNs are smaller than that of SLN, it can even be considered to omit SLNB in combination with the image characteristics, pathological grade and biological behavior of the lesion itself [44]. Because 5–8% of patients still develop lymphedema after the SLNB, and 60% of newly diagnosed breast cancer patients are pathologically lymph node negative, these patients do not benefit from it [45]. Furthermore, Data from Felix Jozsa et al. meta-analysis support the omission of SLNB in clinically and radiographically negative axilla [46]. Due to the object of our study was SLN, we can consider direct ALND for these cases if MDCT-LG indicates skip metastasis when non-SLN metastasis is obvious.

Our study focused only on SLN, but it is still useful for preoperative assessment of ALN status, especially the status of SLN. For those lymph nodes with typical cortical structure, we believe that it may be possible to select patients who can avoid SLNB. Though it is still not a replacement for the SLNB now, some scholars considered that the removal of these LNs is not a necessary procedure to prevent the spread of the disease in recent years and axillary surgery for breast cancer patients has tended to be downgraded [47]. Moreover, we want to emphasize that the images may be affected by uncertainties, and in these cases, fuzzy preprocessing techniques should be used, because it can enhance the contrast of the image, make the image more clear, the measured value will be more accurate [48, 49].

It is hoped that future prospective clinical trials will confirm these results, and that better scanning methods and parameters will be developed to assess ALN and accurately select patients with direct ALND and those who are exempt from SLNB. For those cases that are difficult to determine, the SLNB is performed first and then according to the Z0011 standard. Limitations of the study include: first, there may be biases due to the difficulty in making paired comparisons between MDCT imaging and pathologic findings in each lymph node. Second, this is a retrospective study with a relatively small sample size from a single institution, which may compromise the representativeness of the study.

## Conclusion

SLN mapped by MDCT-LG has the potential to predict ALN metastasis. Cortical thickness of SLN in MDCT-LG was an independent predictor of ALN metastasis. The optimal cut-off value of cortical thickness for predicting metastatic ALN was 3.5 mm. It may possible to avoid SLNB in patients with clinically and radiographically negative axilla.

## Data Availability

The datasets generated or analyzed in the current study are not publicly available because of patient privacy protection, but are available from the corresponding author upon reasonable request.

## Abbreviations

ALN: Axillary lymph node
ALND: Axillary lymph node dissection
MRI: Magnetic resonance imaging (MRI)
MMG: Mammography
MDCT-LG: Multidetector-row computed tomography lymphography
ROC: Receiver operating characteristic
SLN: Sentinel lymph node
SLNB: Sentinel lymph node biopsy
US: Ultrasonography
